# New sensitive method for HEPES quantification in ^68^Ga-radiopharmaceuticals

**DOI:** 10.1186/s41181-020-00093-x

**Published:** 2020-05-14

**Authors:** I. F. Antunes, G. M. Franssen, R. Zijlma, P. Laverman, H. H. Boersma, P. H. Elsinga

**Affiliations:** 1 Department of Nuclear Medicine and Molecular Imaging, University of Groningen, University Medical Center Groningen, Hanzeplein 1, 9713GZ Groningen, The Netherlands; 2 Department of Radiology and Nuclear Medicine, Radboud University Medical Center, Nijmegen, The Netherlands

**Keywords:** ^68^Ga-radiopharmaceuticals, HEPES, HPLC and quality control

## Abstract

**Background:**

The introduction of a GMP-certified ^68^Ga-generator spurred the application of ^68^Ga-radiopharmaceuticals. Several radiosynthesis of ^68^Ga-radiopharmaceuticals are more efficient and robust when performed with 2-[4-(2-hydroxyethyl)piperazin-1-yl] ethanesulfonic acid (HEPES) buffer, which is considered as an impurity in the quality control (QC) procedure. Thus, prior to clinical use, QC must be conducted to ensure that HEPES does not exceed the maximum dose of 200 μg/V _Injected_ as described in *European Pharmacopoeia* (Ph Eur) for edotreotide. However, when applying the thin-layer chromatography (TLC) method described in the Ph Eur to quantify the HEPES amount present in the ^68^Ga-octreotide or in the remaining ^68^Ga-radiopharmaceuticals that were tested, no amount was detectable after 4 min of iodine incubation. Here we tested our modified TLC method and validate a new high-performance liquid chromatography (HPLC) method to quantify HEPES in ^68^Ga-radiopharmaceuticals and compare it to the TLC-method described in Ph Eur. In addition, samples collected from various institutes were tested to evaluate whether the synthesis of different ^68^Ga-radiopharmaceuticals or the use of different synthesis methods could affect the amounts of HEPES.

**Results:**

HEPES could not be detected by the TLC method described in the Ph Eur within 4 min incubation in an iodine-saturated chamber. As for our modified TLC method, only after 2 h, spots were only visible > 1 mg/mL. The HPLC method had a limit-of-quantification (LOQ) of 3 μg/mL and a limit-of-detection (LOD) of 1 μg/mL. From the three ^68^Ga-radiopharmaceuticals tested, only in the [^68^Ga]Ga-NODAGA-Exendin samples exceeding amounts of HEPES were found and its concentration in the [^68^Ga]Ga-NODAGA-Exendin was significantly higher, when compared to [^68^Ga]Ga-DOTATOC and [^68^Ga]Ga-PSMA-11.

**Conclusion:**

The TLC method described in Ph Eur and our modified TLC method may not be sufficiently sensitive and thus unsuitable to use for QC release. The new HPLC method was sensitive, quantitative, reproducible and suitable for QC release. With this method, we were able to determine that some ^68^Ga-radiopharmaceuticals may exceed the HEPES limit of 200 μg/ V _Injected_. This new analytical system would allow correcting for the maximum injected dose in order not to exceed this amount.

## Background

In the last two decades, the development and use of ^68^Ga-based radiopharmaceuticals had continuously grown worldwide. Some of the important reasons for such progress are the availability of GMP-certified ^68^Ge/^68^Ga generators, diverse and robust radiolabelling chemistry and the potential for personalized medicine and for theranostic use (Velikyan 2013).

^68^Ga can be labeled to a wide range of ligands specifically targeting receptors, enzymes, and antigens; small effectors or hapten molecules for pre-targeting imaging and molecules with a biological function to image biological properties and /or processes such as hypoxia, glycolysis, apoptosis, angiogenesis, and proliferation (Velikyan 2015).

^68^Ga-radiopharmaceuticals have been mostly applied in oncology in imaging prostate-specific membrane antigen (PSMA), somatostatin receptors (SSTR), glucagon-like peptide 1 receptors (GLP1R) and many more. However, it has also shown potential for imaging pulmonary or myocardial perfusion as well as infection and inflammation (Velikyan 2015).

During the radiolabelling of ^68^Ga-radiopharmaceuticals, a buffer must be used to assure the correct pH for the incorporation of ^68^Ga radionuclide. The most common buffers used in these labeling are sodium acetate, ammonium acetate and 2-[4-N-(2-hydroxyethyl)-1-piperazinyl]-N′-ethanesulfonic acid (HEPES). Some ^68^Ga radiolabelling reactions have shown to be more efficient and stable when performed with a HEPES buffer solution (Sasson et al. 2010).

HEPES is a zwitterionic compound that is widely used in cell culture due to its higher capacity to maintain physiological pH despite the increasing contents of CO_2_ produced by cellular respiration. HEPES is considered to be an impurity and at present, information regarding HEPES toxicity is lacking. Therefore, despite being considered eligible for human use, doubts still remain regarding its safety in humans (Meyer et al. 2004). The European Pharmacopeia (Ph Eur) has considered the HEPES present in ^68^Ga-radiopharmaceuticals as an impurity which should not exceed the maximum dose of 200 μg/ V _Injected (_01/2013:2482 Gallium (68Ga) Edotreotide injection 2011_)_. The Ph Eur has included a TLC method for the detection of HEPES which should be performed prior to release. However, when applying this TLC method to detect the HEPES present in the ^68^Ga-radiopharmaceuticals within the 4 min incubation with Iodine, no spot was visible.

This led us to test a modified TLC method to see if detection of the HEPES would improve and in addition, to develop a new HPLC method to quantify the HEPES amount present in solutions (Antunes et al. 2017). Here we compare the TLC methods with a faster and reliable quantitative HPLC method to determine the amounts of HEPES present in the final formulated ^68^Ga-radiopharmaceuticals.

## Methods

### Reagents and solvents

^68^Ga synthesis kits and reagents were obtained from Advanced Biochemical Compounds (ABX, Radeberg, Germany), ammonium formate, iodine beads, and HEPES were obtained from Sigma-Aldrich. Silica gel 60 F245 (20 × 20 cm) TLC plates were purchased from Merck.

### ^68^Ga-radiopharmaceuticals

For ^68^Ga, a > 9 month old GMP 1110 MBq grade ^68^Ga-generator was used (Eckert & Ziegler). Radiopharmaceuticals were synthesized in compliance with each institute’s GMP protocol. The syntheses were performed using a GRP® synthesis module from Scintomics (Fürstenfeldbruck, Germany) with the cationic concentration protocol provided by Scintomics. Briefly, the [^68^Ga]GaCl_3_ was concentrated on a PS-H^+^ cartridge and eluted with a 5 M NaCl solution into a reaction vial containing either 5–10 μg PSMA-11 or 38 μg DOTATOC, in 2 mL of HEPES 1.5 M buffer. The reaction mixture was allowed to react for 10 min at 100 °C. After cooling down, the mixture was trapped on a Sep-Pak C18 light (pre-activated with EtOH and WFI) and subsequently eluted with 2 mL of a mixture of EtOH/water (1:1) and diluted in 16 and 18 mL of PBS, respectively.

The radiolabelling of [^68^Ga]Ga-NODAGA-Exendin was performed according to the procedure described in literature (Franssen et al. 2015). Briefly,[^68^Ga]GaCl_3_ was eluted from the PS-H^+^ cartridge (pre-activated with 5 M NaCl in HCl 0.1 M and WFI) with NaCl 5 M in 0.1 M HCl and allowed to react with 10 μg NODAGA-Exendin in 475 μL of HEPES 2.5 M and 50 μL of ascorbic acid (100 mg/ml), for 10 min at 95 °C. The mixture was cooled down, 2.0 ml 50 mM EDTA, 0.15% Tween-80 was added and passed through an HLB Plus light cartridge (pre-activated with EtOH and WFI). After washing the cartridge with water, the final product was eluted with 1 mL of ethanol and diluted with 19 mL of PBS.

All formulated products were sterilized by filtration over a 0.22 μm filter into a sterile vial. The final products were released by quality control (QC) after routine tests.

### TLC analysis

#### TLC analysis Ph Eur. method

The TLC analysis was performed according to the Ph Eur. The reference solution was prepared as described (01/2013:2482 Gallium (68Ga) Edotreotide injection 2011). Briefly, 10 mg of HEPES was dissolved in 10 mL water. From this solution, 1 mL was diluted to 50 mL with water, resulting in a HEPES concentration of 0.02 mg/mL. From this 0.02 mg/mL HEPES solution, 1 μL was spotted on a TLC silica gel F_254_ plate and dried with a current of air. Once the spots was dried and not visible anymore, the TLC plate was eluted with a solution of water/acetonitrile (25/75 v/v) as the mobile phase. The elution was stopped when the front of the mobile phase reached 2/3 of the plate. The plate was then dried and transferred to the iodine vapor chamber and incubated for 4 min. After incubation, the plate was immediately scored for a positive signal (a clear visible yellow spot corresponding to HEPES).

#### TLC analysis modified Ph Eur. method

The TLC analysis was performed according to the Ph Eur with minor modifications. HEPES solutions with different concentrations ranging from 1 to 0.006 mg/mL were prepared, using the matrix solution of the formulated samples (10% EtOH in PBS). From each HEPES solution a total amount of 10 μL was spotted on 4 TLC silica gel F_254_ plate (one for eact time of incubation). The volume of 10 μL was spotted in five steps of 2 μL and dried with a gentle stream of air in-between. Once the spots were dried and not visible anymore, the TLC plates were incubated in a TLC container and eluted with a solution of water/acetonitrile (25/75 v/v) as the mobile phase. The incubation was stopped when the front of the mobile phase reached 2/3 of each plate. The plates were dried and transferred to the iodine vapor chamber and incubated for 4 min according to the Ph Eur and 1, 2 or 24 h. After incubation, the plates were immediately scored for a positive signal (a clear visible yellow spot) for any of the spotted HEPES concentrations. This experiment was performed in triplicate.

### HPLC analysis

HEPES solutions with concentrations ranging from 6 to 100 μg/mL were prepared with the matrix solution of the formulated samples (10% EtOH in PBS). HPLC analysis was performed with a Waters Acquiry QSm (quaternary solvent manager) pump system using a Waters XBridge C18 column (150 mm × 4.6 mm, 5 μm), connected to a Waters Acquiry 4-class UV detector set to a wavelength of 195 nm. The mobile phase consisted of ammonium formate 20 mM pH 9.5, at a flow rate of 1 mL/min). Chromatograms were collected and analyzed with Empower 3 software® (Waters). Each sample was tested in triplicate unless stated otherwise. The HPLC method was validated according to the ICH Q2 (R1) guidelines (Borman and Elder 2017).

#### Calibration curve

Seven HEPES solutions with concentration ranging from 6 to 100 μg/mL were used for each calibration curve. Each of these solutions was injected in triplicate and in 3 consecutive days. The final calibration curve was from the average of the 3 calibration curves obtained in those 3 consecutive days.

#### Accuracy

The accuracy of the HPLC method was evaluated in triplicate at seven concentrations ranging from 6 to 100 μg/mL. The accuracy value is expressed as the ratio between the value of the determined HEPES concentration and the original standard know concentration.

#### Inter-day repeatability

*Three solutions with different concentrations of HEPES (Low = 8* μg/mL*; medium = 15* μg/mL *and high = 50* μg/mL) were injected five times and in three consecutive days. An F-test was applied to evaluate if there were any differences in the area of the peaks obtained in each day.

#### Intra-day repeatability

For the intra-day analysis, we choose the solution with 15 μg/mL (medium concentration) which was the closest to the concentration we would perform our analysis (20 μg/mL). This solution (15 μg/mL) was analyzed five times in the same day. The intra-day repeatability is expressed as a relative standard deviation (RSD%).

#### Limit-of-detection (LOD) and limit-of-quantification (LOQ)

The LOD and LOQ for HEPES were determined at signal-to-noise ratios of 3:1 and 10:1, respectively.

### Revalidation of the methods

**Fig. 1:**
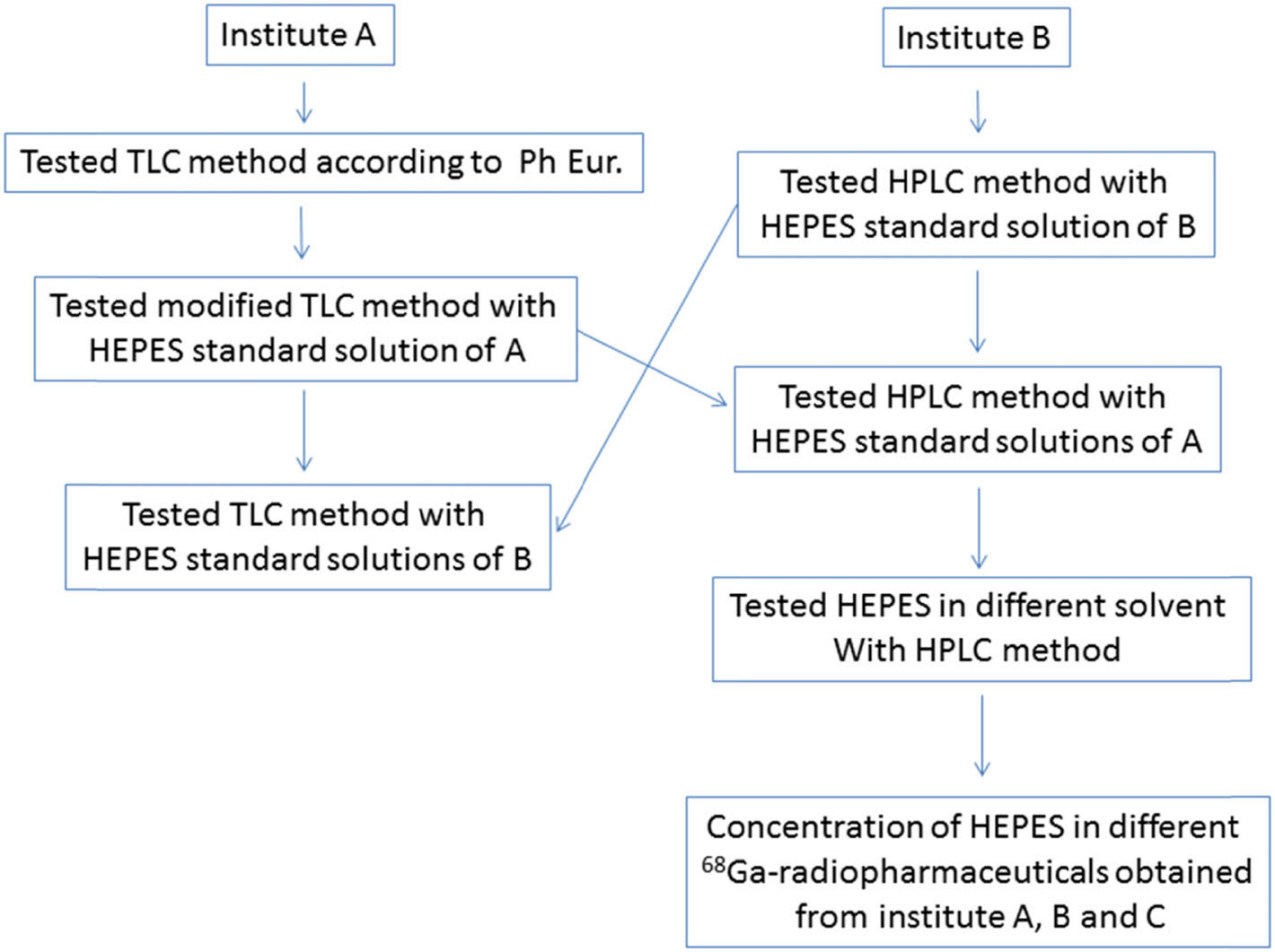
Scheme of the setup of the performed experiments

Standard HEPES samples ranging from 6 to 100 μg/mL prepared in the two different institutes were exchanged to the other institute to be tested either with the TLC (modified method) or the HPLC method depending of the institute (Fig. 1).

### Solvent effect

HEPES samples (10 μg/mL) were prepared in either 10% (v/v) ethanol in PBS, PBS or NaCl 0.9% to evaluate the matrix effect in the determination of HEPES in solution by the HPLC method (Fig. 1). The experiment was performed in triplicate.

### Determination of HEPES in different ^68^Ga-radiopharmaceuticals

**Fig. 2:**
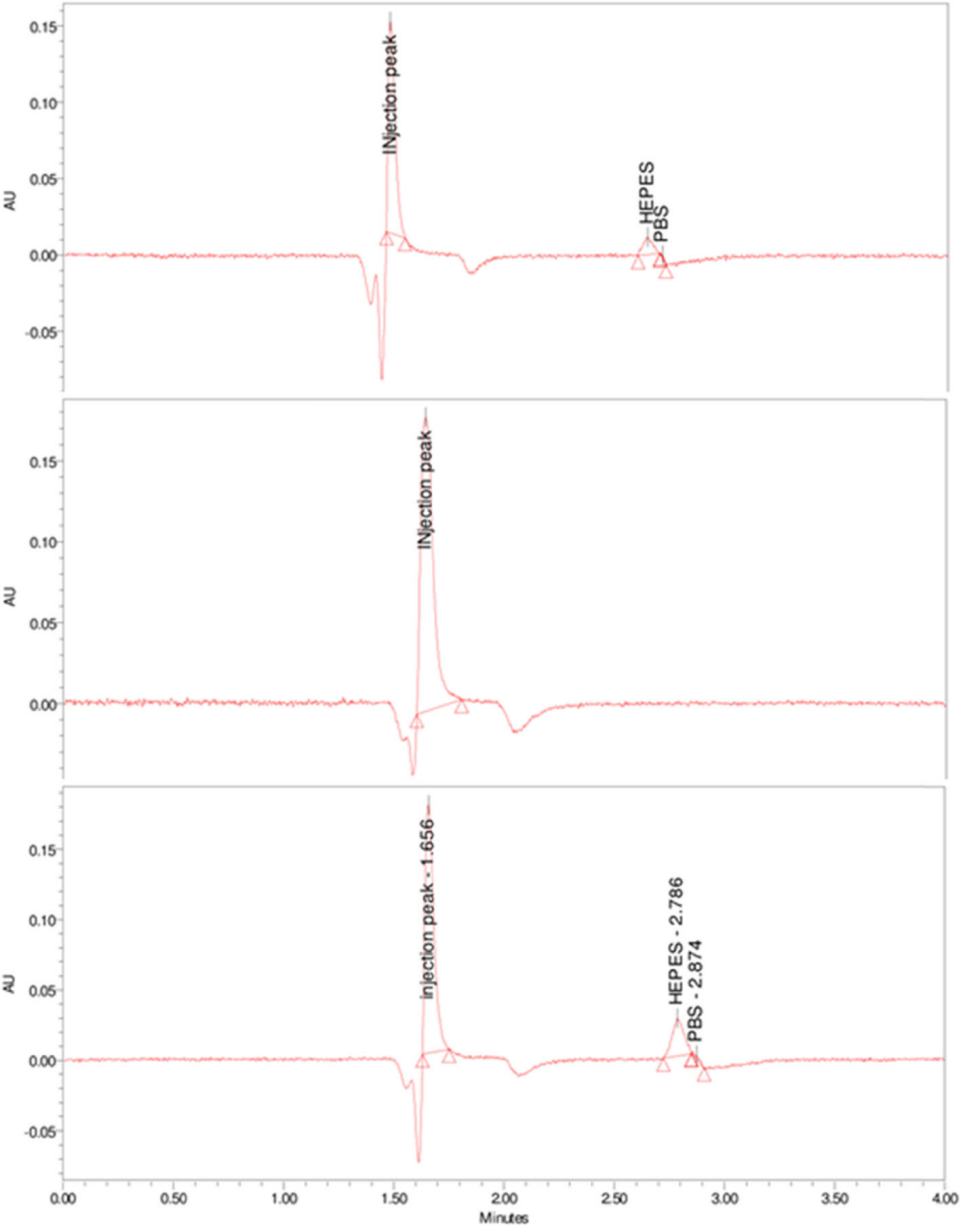
Examples of chromatograms with an isocratic system of NH_4_HCO_2_ 20 mM pH 9.5, UV 195 nm, from 0 to 4 min of: an HEPES solution (top); a GA-DOTATOC (middle) solution and a [^68^Ga] GaDOTATOC solution (bottom)

Samples of [^68^Ga]Ga-DOTATOC; [^68^Ga]Ga-PSMA-11 and [^68^Ga]Ga-NODAGA-Exendin from different batches, for clinical use, were collected from different institutes and tested in the institute where the HPLC method was developed. To assure that the HEPES peak was not altered by the peak of the radiopharmaceutical, an injection of the radiopharmaceutical without HEPES was performed (Fig. 2).

### Statistics

Statistical analyses were performed with Excel 2010 (Microsoft) and GraphPad Prism (version 7.02). Inter-day repeatability was analyses with an F-test. Differences in solvent effects were analyzed using a two-sided unpaired Student’s t-test. Correlations were calculated with the linear regression algorithm in GraphPad Prism (version 7.02) and were considered statistically significant whenever r^2^ > 0.5 and *p* < 0.05. The revalidation of the method was performed with a Pearson comparison. Differences of HEPES found in different ^68^Ga-radiopharmaceuticals were analyzed by 1-way ANOVA multiple comparisons. Significance was reached when the probability (p) value was ≤0.05. Throughout the manuscript, values are presented as mean ± the standard deviation (SD).

## Results

### TLC analysis

The HEPES reference sample of 0.02 mg/ml in water was prepared as described in the Ph Eur. After 4 min incubation in iodine vapor, no bright yellow spot was visible (data not shown).

In the modified TLC method, after 4 min incubation (according to the Ph Eur) in the iodine vapor chamber, none of the HEPES samples showed a (bright) yellow signal. Moreover, incubation for 1 h in the iodine vapor chamber did also not result in a clear signal for any of the spotted HEPES concentrations. Only after incubation of 2 h, the 1 mg/mL HEPES sample showed a yellow signal. This signal became more intense after 24 h incubation. Nevertheless, HEPES concentrations < 0.1 mg/mL did not show any signal even after 24 h.

### HPLC analysis

To validate the HPLC method a calibration curve was generated from an average of three measurements containing 7 different concentrations of HEPES (Fig. 4). The correlation coefficient of the calibration curve was *r*^2^ = 0.997 between the HEPES concentration and the peak area in the range of 6–100 μg/mL. The HPLC method had an inter-day (five replicate determinations in three consecutive days) reproducibility ranging from 93 to 107% with a relative standard deviation (RSD) of 2.26% and an intra-day (five replicate determinations in the same day) reproducibility ranging from 96 to 102% within an RSD of 2.30%, respectively. The accuracy was found to be 96 ± 0.12% with a LOQ and a LOD of 3.23 and 0.97 μg/mL, respectively (Table 1).

**Table 1:** Parameters required for the validation of the HPLC method

	Requirements	Results
**Retention time**	< 4 min	2.8 min
**Intra-day**		
**Repeatability**	RSD < 5.00%	2.30%
**Noise**	> 10	43
**Inter-day**		
**Reproducability**	F-test> 0.05	F-Test > 0.65
**Linearity UV-detector**	R^2^ > 0.98	*R*^2^ = 0.99
**LOD**	3x noise	2.74 ± 0.24
**LOQ**	10x noise	9.40 ± 1.25
**Carry over**	≤0.10%	0.08%

### Solvent effect

The intensity of HEPES peaks, when dissolved in different solutions, was found to be not significantly different from each other (Table 2). However, the use of a matrix containing 10% Ethanol in PBS shifted the retention time from 2.70 min to 2.60 min.

**Table 2:** Effect of the solvent in the quantification of HEPES. Each solution was analyzed by HPLC in triplicate

Solvent	Retention time (min)	Concentration of HEPES (μg/mL)	t-test (p)
Matrix (10% EtOH/PBS)	2.60 ± 0.01	9.35 ± 0.16	____
NaCl 0.9%	2.69 ± 0.01	9.35 ± 0.29	0.99
PBS	2.70 ± 0.01	9.98 ± 0.40	0.09

### Revalidation of the methods

**Fig. 3:**
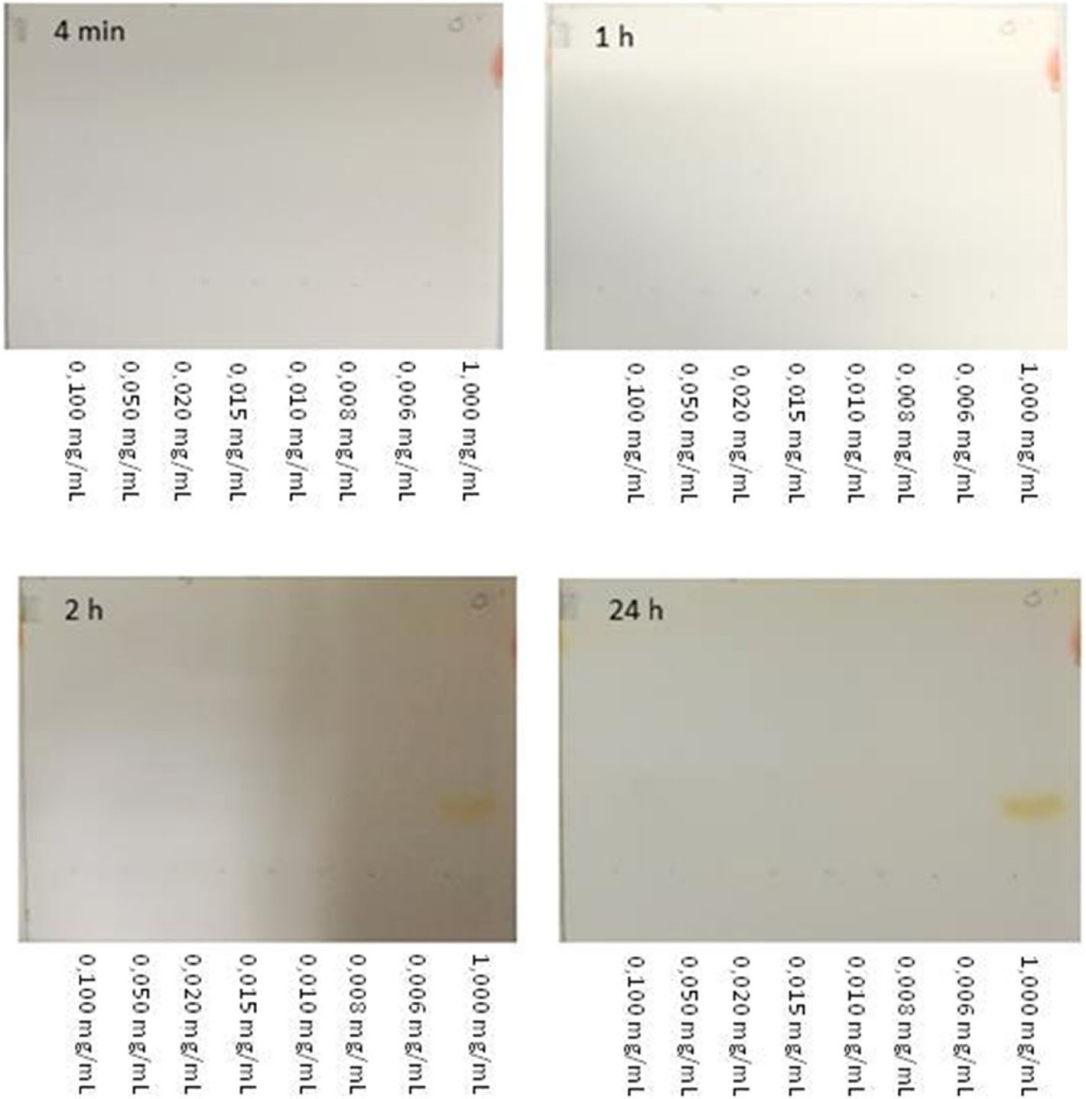
TLC of HEPES incubated in iodine chamber for 4 min; 1 h; 2 h and 24 h

**Fig. 4:**
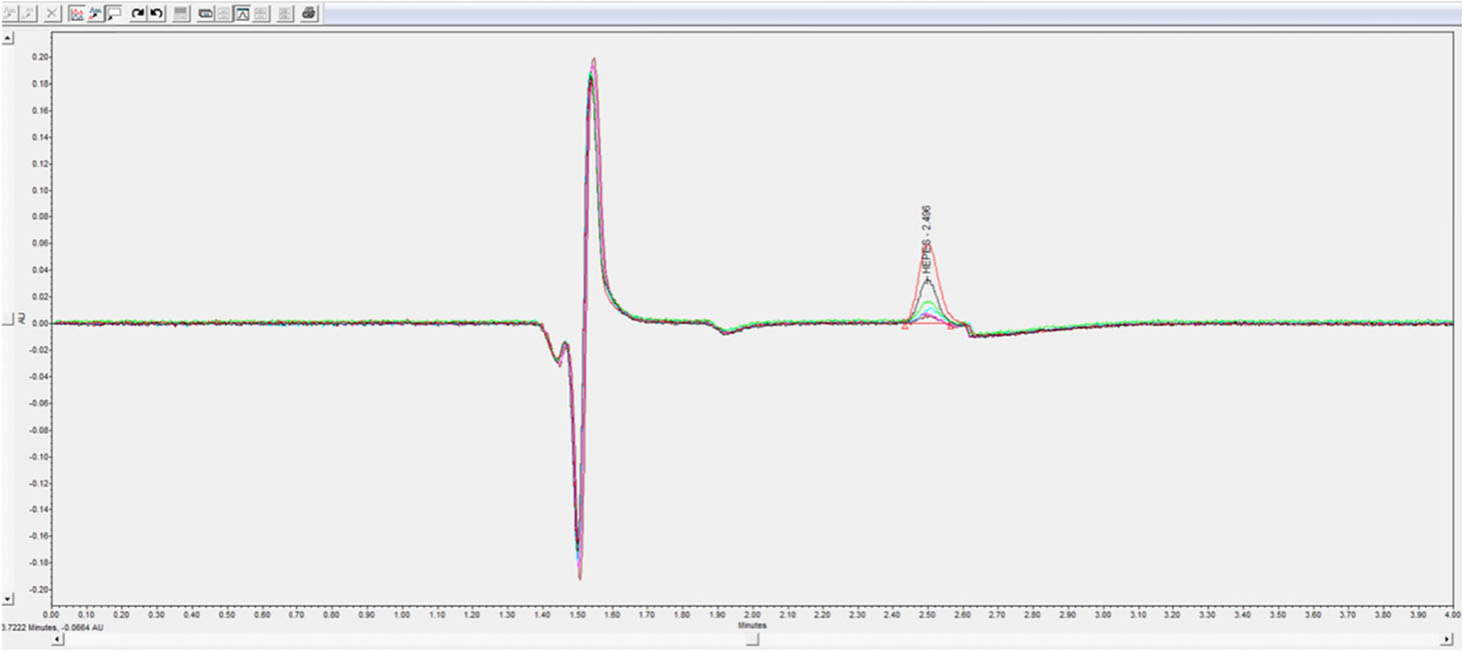
Chromatograms of HEPES in the range of 6–100 μg/mL with an isocratic system of NH_4_HCO_2_ 20 mM pH 9.5, UV 195 nm, from 0 to 4 min

**Fig. 5:**
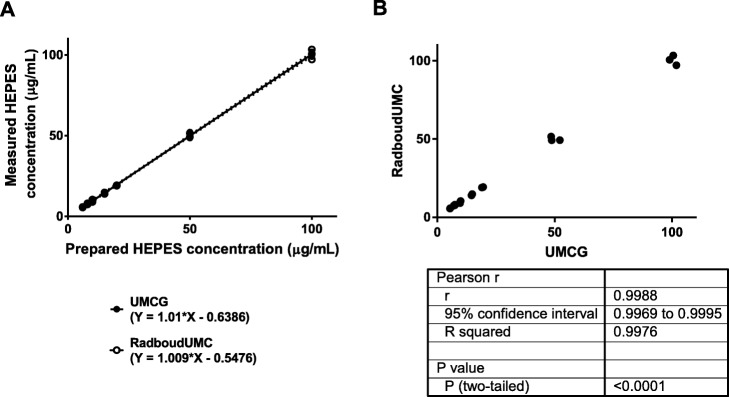
**a** Calibration curves obtained with solutions prepared in two different institutes. **b** Comparison of the 2 calibration curves

The TLC analysis performed with the solutions form institute B revealed once again that none of the diluted concentrations were visible within any time frame (Fig. 3). Only the stock solution of 1 mg/mL was clearly visible 2 h and 24 h after iodide incubation. For the HPLC comparison (Fig. 4), standard HEPES solutions prepared at two institutes were used to generate two calibration curves with linearity of *r*^2^ = 0.9993 and 0.9988 between the HEPES concentration and the peak area in the range of 6–100 μg/mL (Fig. 5a). The Pearson’s correlation coefficient of *r* = 0.9988 (Fig. 5b) confirmed the significant relationship between both calibration curves.

### HEPES analysis in different ^68^Ga-radiopharmaceuticals

The HEPES concentrations in the final formulations of [^68^Ga]Ga-DOTATOC, [^68^Ga]Ga-PSMA-11 and [^68^Ga]Ga-NODAGA-Exendin that were analyzed by the HPLC method are stated in Table 3.

**Table 3:** Amounts of HEPES in different ^68^Ga-radiopharmaceuticals obtained from different institutes and analyzed by the HPLC method (*n* = the number of samples from different batches)

	[^68^Ga]Ga-DOTATOC		
	HEPES (μg/mL)	Maximum amount in V_Inj_ (μg) ^a^	Maximum V_Inj_ (mL)
**Institute A** (*n* = 3)	21 ± 4	171 ± 35	10 ± 2
**Institute B** (*n* = 6)	19 ± 8	150 ± 60	11 ± 1
**Institute C**	____	____	____
	[^68^Ga]Ga-PSMA-11		
**Institute A** (*n* = 3)	7 ± 10	58 ± 81	14 ± 3^b^
**Institute B** (*n* = 5)	8 ± 7	64 ± 54	16 ± 3^b^
**Institute C**	____	____	____
	[^68^Ga]Ga-NODAGA-Exendin		
**Institute A** (*n* = 3)	31 ± 1^§^	291 ± 39	6 ± 1
**Institute B** (*n* = 3)	36 ± 5^§^	245 ± 12	6 ± 1
**Institute C** (*n* = 3)	60 ± 8*	482 ± 69	2 ± 1

^a^ When considering the recommended volume of injection of 8 mL. ^b^ When the amounts of HEPES were below LOQ, the total volume of the radiopharmaceutical (V_T_ = 16 mL) was considered the maximum injected volume. ^§^*p* < 0.05 when comparing the HEPES concentration present in different radiopharmaceuticals obtained from the same institute.* *p* < 0.05 when comparing the HEPES concentration present in the same radiopharmaceutical obtained in different institutes

## Discussion

Currently, only edotreotide ([^68^Ga]Ga-DOTATOC) radiolabeling is included in the Ph Eur. In this procedure, HEPES is considered as an impurity and therefore a TLC method for the detection of HEPES in [^68^Ga]Ga-DOTATOC is described. In the present study, we describe a new TLC method and a sensitive HPLC method for the quantification of HEPES present in ^68^Ga-radiopharmaceuticals. This HPLC method is more reliable than the TLC assay described in Ph Eur. In addition, we also could not detect the maximum amount stated by the Ph Eur (200 μg/volume of injected sample (10 mL) e.g. 20 μg/mL) in the modified TLC method. In fact, in the modified TLC method, the HEPES spots were only visible at concentrations higher than 1 mg/mL after 2 h of iodine incubation instead of 4 min as stated in the Ph Eur. Thus, this modified TLC method, as well as the method recommended by the Ph Eur, may not be suitable to use for a release requirement. With the new HPLC method, we were able to have an LOQ of 3.2 μg/mL. These results are in similar range with a previously described HPLC method where the LOQ was 10 μg/mL^3^ (Sasson et al. 2010).

With this new HPLC method, it was found that the presence of various solvents did not affect the quantification of HEPES. This makes the method particularly useful when various ^68^Ga-radiopharmaceuticals are formulated with different solutions. In addition, with a retention time of 2.60–2.80 min it allows the QC to be performed within 4 min and therefore suitable when a pre-release is required, as described in the Ph Eur. The method is highly reproducible since we showed that when measuring a similar solution, prepared by two different institutes, no significant differences were found between both measurements (Fig. 5).

From the different ^68^Ga-radiopharmaceuticals that were analyzed, it was found that only the amounts of HEPES present in [^68^Ga]Ga-NODAGA-Exendin were significantly higher than the amounts found in the [^68^Ga]Ga-DOTATOC and [^68^Ga]Ga-PSMA-11 synthesized in the same institute. This difference is most likely due to the different labeling method used for [^68^Ga]Ga-NODAGA-Exendin.

In routine, in principle, a limit test to determine if the HEPES content is below the maximum limit would be sufficient. Thus, the more reliable TLC method developed by Pfaff et al. could be used in any nuclear medicine center (Pfaff et al. 2018). However, even this method may sometimes give an overestimation of the HEPES amounts present in a sample, risking the non-release of the radiopharmaceutical. Therefore, a quantitative method like the HPLC would provide information not only of the total HEPES amount present in the radiopharmaceutical but also, in case of the HEPES amount is above the maximum limit, it can calculate the maximum volume that the technician can take in order not to exceed the maximum limit, avoiding not releasing the radiopharmaceutical. A clear example of this situation can be observed with the [^68^Ga]Ga-NODAGA-Exendin. In the presence of the TLC methods only, none of the [^68^Ga]Ga-NODAGA-Exendin could be released since their amount of HEPES exceeds the maximum limit. However, with the HPLC method, as long as the activity is high enough, a smaller volume of the tracer could be injected in order not to exceed the maximum amount of HEPES (Table 3). One of the limitations of the HPLC method is the need for an extra HPLC if the test needs to be performed before the release of the radiopharmaceutical. Thus, the HPLC method can only be introduced in centers where there are at least 2 HPLC systems. In case this is not possible the TLC method developed by Pfaff et al. could be implemented since they showed similar results obtained with their TLC method compared to our HPLC method.

## Conclusion

The TLC method described in European Pharmacopoeia proved to be insufficiently sensitive and thus unsuitable to use for QC release. The herewith presented HPLC method is sensitive, quantitative, reproducible and suitable for QC release.

## Data Availability

The datasets used and/or analyzed during the current study are available from the corresponding author on reasonable request.

## Abbreviations

HEPES: 2-[4-(2-hydroxyethyl)piperazin-1-yl] ethanesulfonic acid
TLC: Thin layer chromatography
HPLC: High-performance liquid chromatography
Ph Eur: European Pharmacopeia
LOQ: limit-of-quantification
LOD: limit-of-detection
QC: quality control
GMP: Goog manufacturing practice
PBS: Phosphate buffer saline
